# Supplementary material for: Hyperthermia-triggered biomimetic bubble nanomachines
Source: Nat Commun. 2023 Aug 11;14:4867. doi: 10.1038/s41467-023-40474-9 (PMC10421929; doi:10.1038/s41467-023-40474-9)
Supplement: Supplementary file 1 — Supplementary Information [file 41467_2023_40474_MOESM1_ESM.pdf]

Supplementary Information

Title:

Hyperthermia-triggered Biomimetic Bubble Nanomachines

Authors: Junbin Gao[1], Hanfeng Qin[1], Fei Wang[1], Lu Liu[1], Hao Tian[1], Hong Wang[1], Shuanghu Wang[2], Juanfeng Ou[1], Yicheng Ye[1], Fei Peng[3, *] and Yingfeng Tu[1, *]

Affiliations:

[1]NMPA Key Laboratory for Research and Evaluation of Drug Metabolism & Guangdong Provincial Key Laboratory of New Drug Screening, School of Pharmaceutical Sciences, Southern Medical University, Guangzhou, 510515, China.

[2]The Laboratory of Clinical Pharmacy, The Sixth Affiliated Hospital of Wenzhou Medical University, The People's Hospital of Lishui, Lishui 323020, China

[3]School of Materials Science and Engineering, Sun Yat-Sen University, Guangzhou, 510275, China.

*Corresponding authors:

Fei Peng, Ph.D.

School of Materials Science and Engineering, Sun Yat-Sen University, Guangzhou, 510275, China.

Yingfeng Tu, Ph.D.

NMPA Key Laboratory for Research and Evaluation of Drug Metabolism & Guangdong Provincial Key Laboratory of New Drug Screening, School of Pharmaceutical Sciences, Southern Medical University, Guangzhou, 510515, China

E-mail: F.P. (pengf26@mail.sysu.edu.cn) or Y.T. (tuyingfeng1@smu.edu.cn).

Supplementary Figures

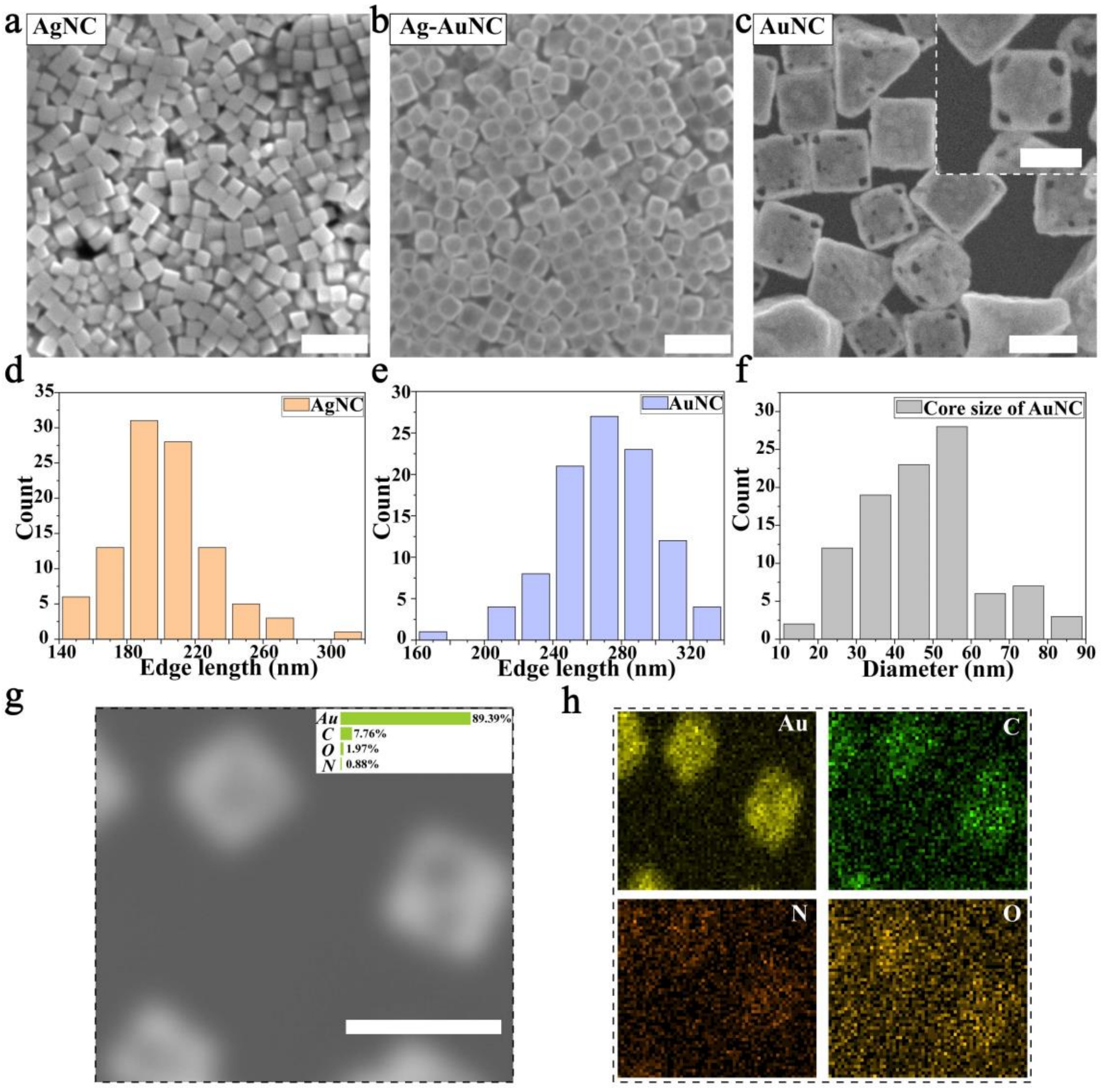

Supplementary Figure 1 Characterization of Ag/Au precursors. SEM images of (a) AgNC, (b) Ag-AuNC and (c) AuNC. Scale bars for (a-b) =1 μm. Scale bar for (c) =200 nm. (d-f) Size distribution of their corresponding nanoparticles (over 100 nanoparticles were counted for statistical analysis). (g) SEM image (inset: atomic percentage of the corresponding nanoparticles) and (h) EDX mapping of AuNC. Scale bar = 500 nm. Experiments were performed three times (a-c) or twice (g-h), with similar results. The inside abbreviations [Au] = Gold, [C] =Carbon, [N] =Nitrogen, [O] =Oxygen.

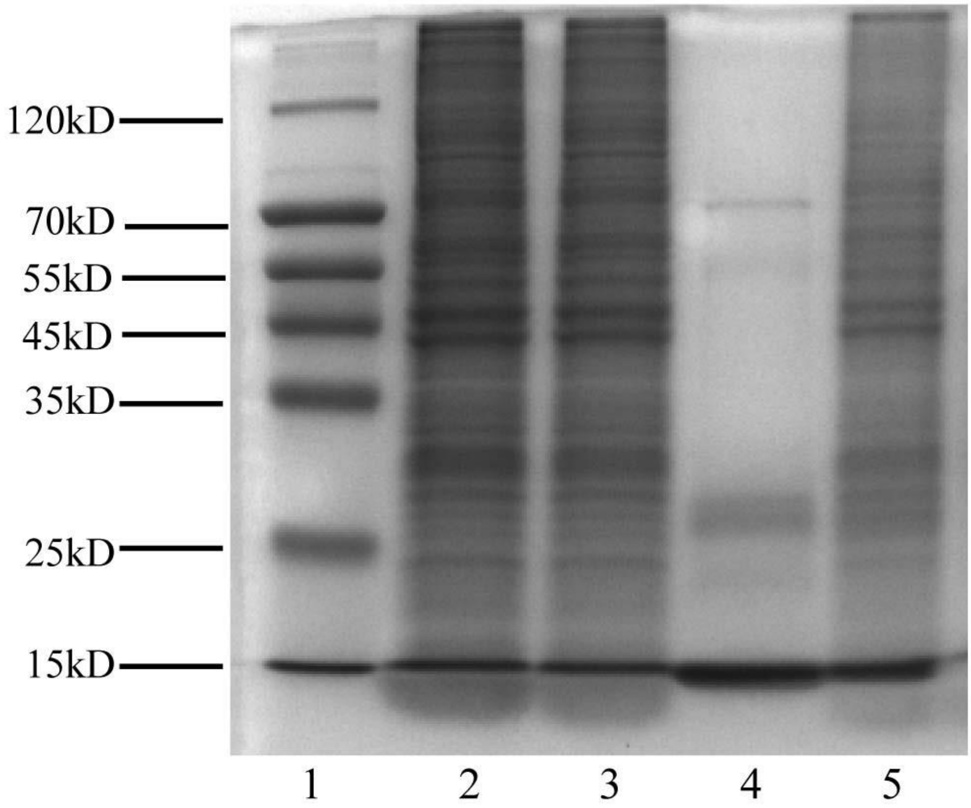

Supplementary Figure 2 Protein analysis of cloaked cell membrane. SDS-PAGE protein analysis (1: marker; 2: 4T1 cell membrane; 3: ICG@CCM-AuNC-PO_2-Hb; 4: Hb; 5: secreted CCM nanovesicles). Experiments were performed three times, with similar results.

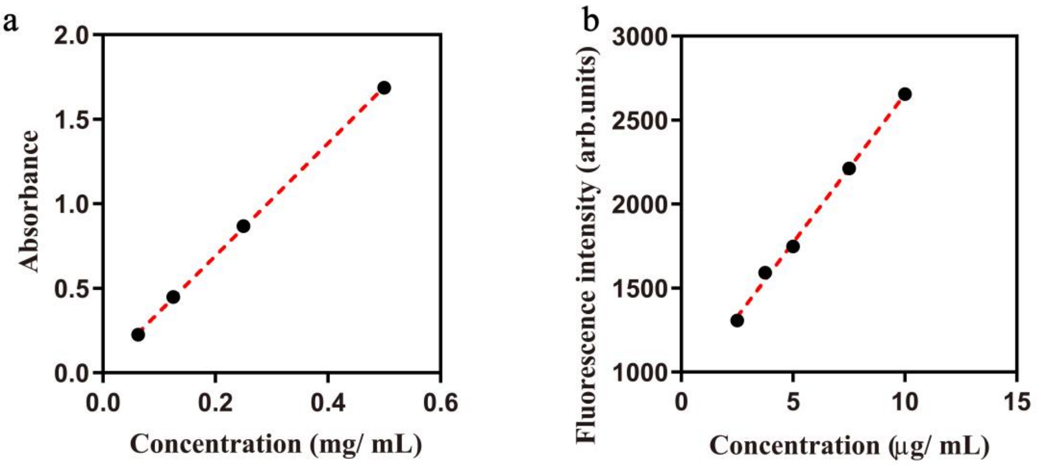

Supplementary Figure 3 The linear relationship of loading materials. (a) Standard curve of Hb. (b) Standard curve of ICG.

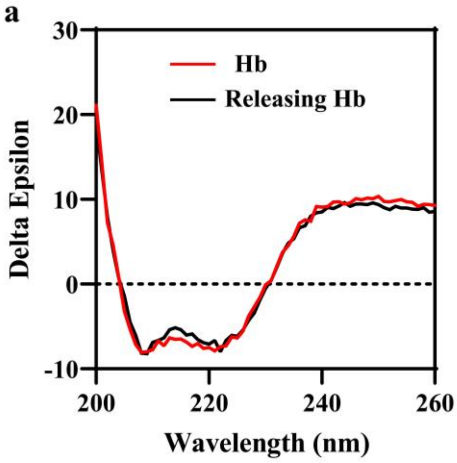

b

	α-helix	β-sheet	Turn	Random
Hb	66.5	8.7	11.4	13.5
Releasing Hb	66.3	8.8	11.4	13.6

Supplementary Figure 4 Stability of hemoglobin. (a) Far-UV CD spectra of Hb or released Hb. (b) Secondary structures of Hb computed by DichroWeb online analysis.

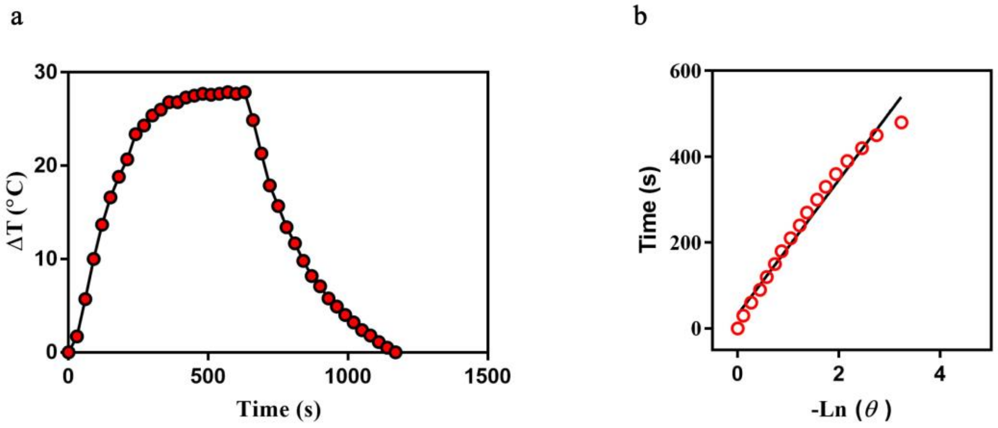

Supplementary Figure 5 Evaluation of photothermal conversion. (a) Photothermal response of ICG@CCM-AuNC-PO_2-Hb aqueous dispersion (200 μg mL^{-1}, 0.2 mL) under NIR irradiation (808 nm, 1 W cm^{-2}). The laser was shut off until once the temperature reached a steady-state value. (b) Linear time data versus –lnθ obtained from the cooling period of Figure S5a.

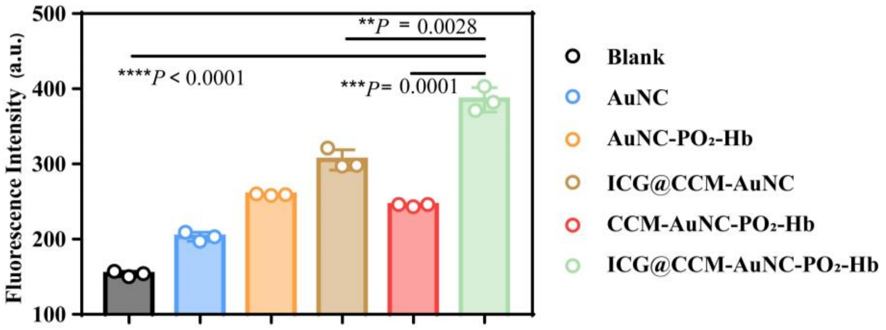

Supplementary Figure 6 Generation of ROS *in vitro*. $^1O_2$ generation of different formulation under NIR irradiation 808 nm (1.0 W cm^{-2}, 2 min). $n = 3$ biologically independent samples. Data are presented as means ± SD. Significant differences were evaluated by two-tailed unpaired t-test. Statistical significance: **P<0.01, ***P<0.001, ****P<0.0001.

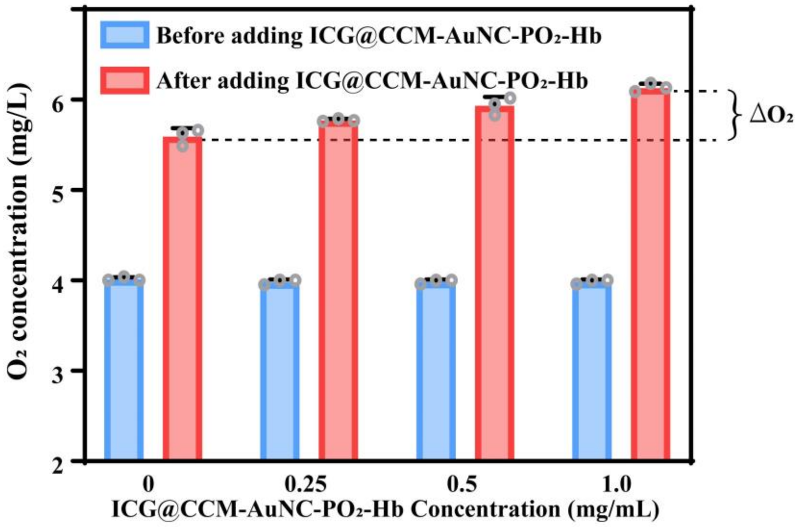

Supplementary Figure 7 Load efficiency of oxygen on the carrier. Oxygen concentration before and after adding 0.2 mL of ICG@CCM-AuNC-PO_2-Hb with different concentrations. ΔO_2: increased O_2 concentration. Oxygen loading capacity per 1 g of ICG@CCM-AuNC-PO_2-Hb was calculated as follows: The loading capacity of ICG@CCM-AuNC-PO_2-Hb = (ΔO_2: increased O_2 concentration) / (ICG@CCM-AuNC-PO_2-Hb concentration). $n = 3$ independent experiments. Data are presented as means ± SD.

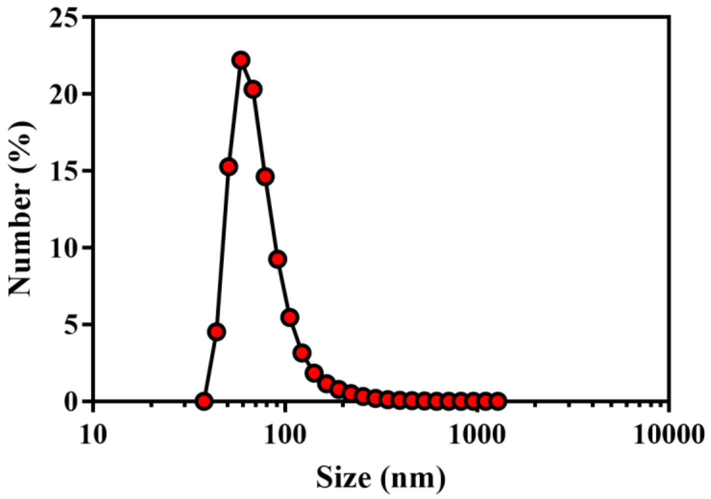

Supplementary Figure 8 Size distribution (number) of hyperthermia-triggered nanovesicles.

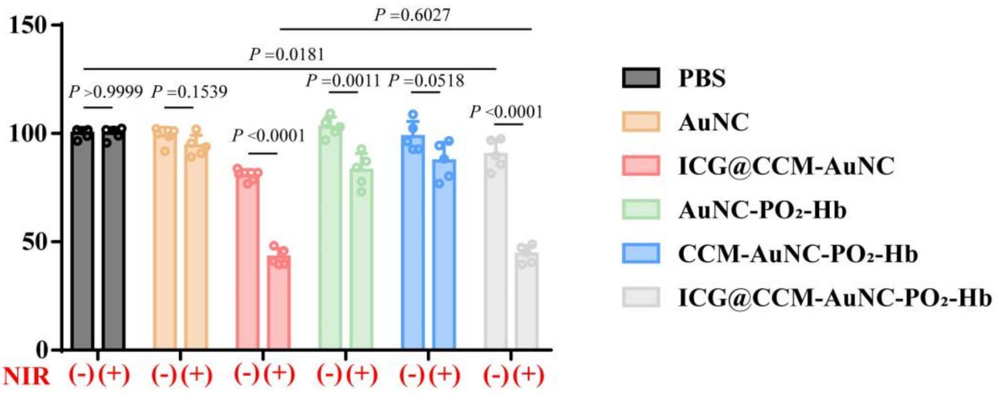

Supplementary Figure 9 Dark/phototoxicity on NIH3T3 cells. Dark viability and phototoxicity of NIH3T3 cells after different treatments. Data are given as means ± SD ($n = 5$ biologically independent samples). Significant differences were evaluated by two-tailed unpaired t-test.

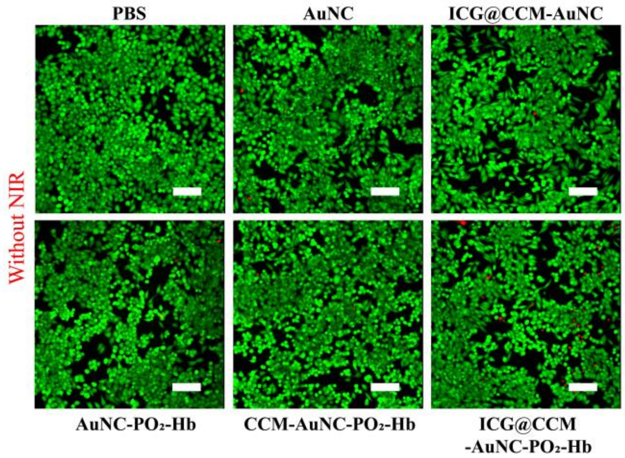

Supplementary Figure 10 Dark viability on 4T1 cells. Fluorescent microscopy images of live/dead staining of 4T1 cells after different treatments without NIR laser irradiation (scale bar = 100 μm). Experiments were performed three times, with similar results.

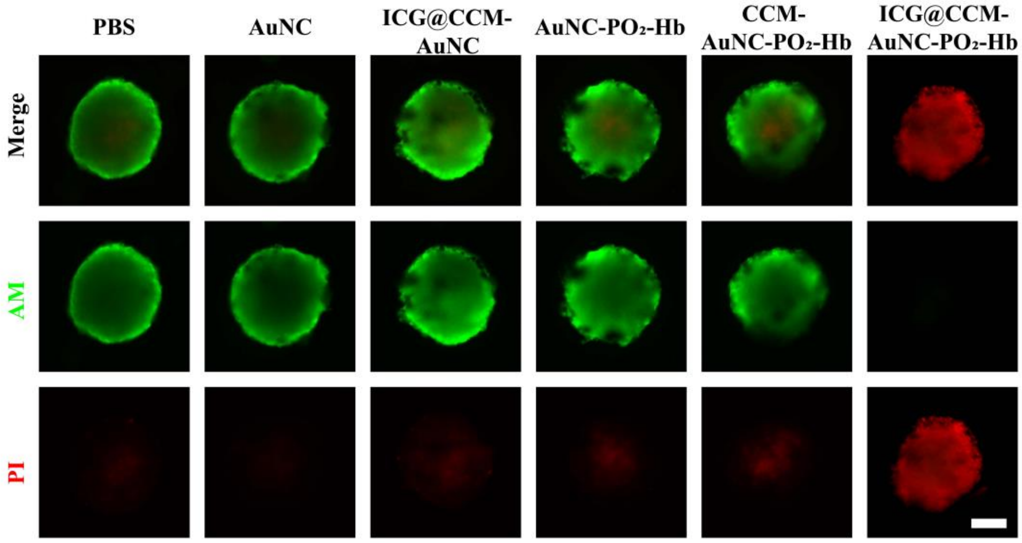

Supplementary Figure 11 Anti-cancer effects on tumor spheres. Live/dead staining of tumor spheroids after incubating with ICG@CCM-AuNC-PO_2-Hb followed by NIR irradiation for 3 min (1.0 W cm^{-2}). Scale bar = 100 μm.

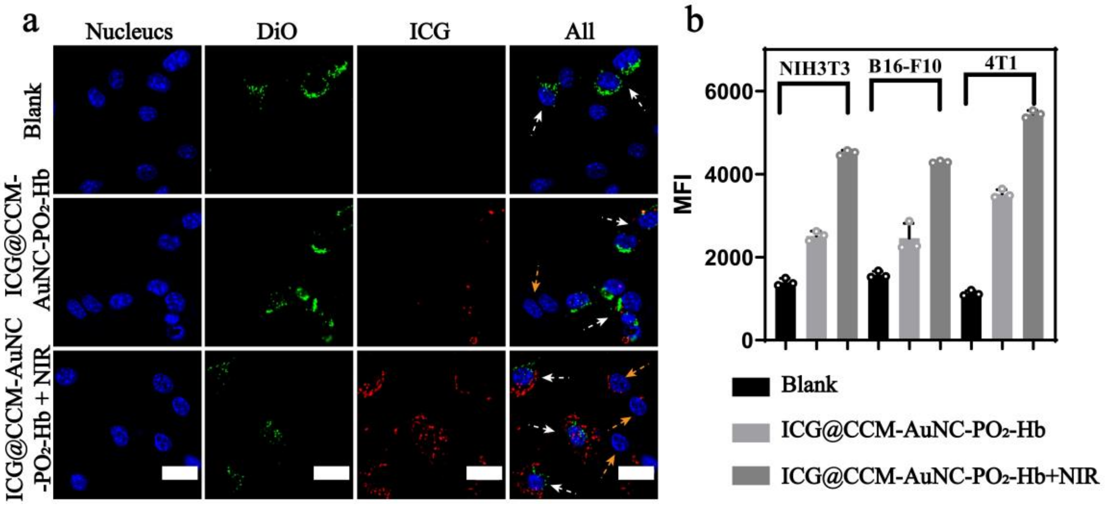

Supplementary Figure 12 Homologous targeting on different cells. (a) Fluorescent imaging of co-cultured 4T1/B16 cells treated with ICG@CCM-AuNC-PO_2-Hb (blue = nucleus, green = 4T1 cells membrane, red = ICG@CCM-AuNC-PO_2-Hb. Scale bar = 30 μm). The white arrows indicated B16 cells and the orange arrows indicated 4T1 cells. (b) Flow cytometry analysis of cells after incubating with ICG@CCM-AuNC-PO_2-Hb. $n = 3$ biologically independent samples. Data are presented as means ± SD. Experiments were performed three times (a), with similar results.

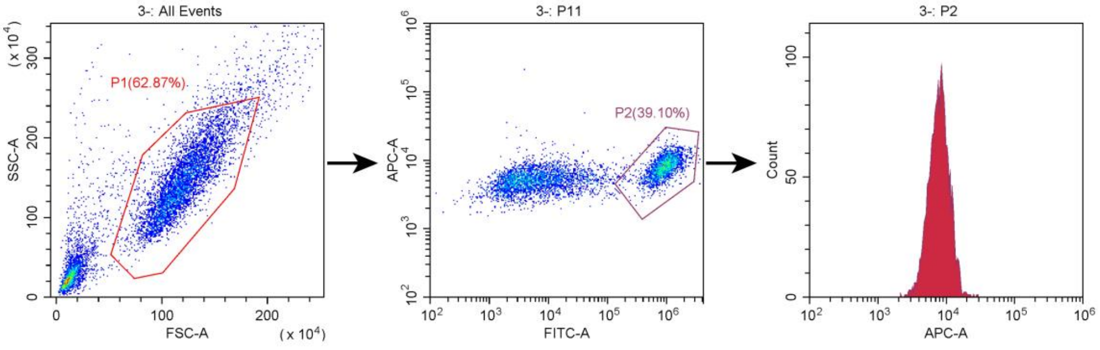

Supplementary Figure 13 Representative scatter plots and gating information derived from homologous targeting analysis of 4T1 cells, B16-F10 cells and NIH3T3 cells.

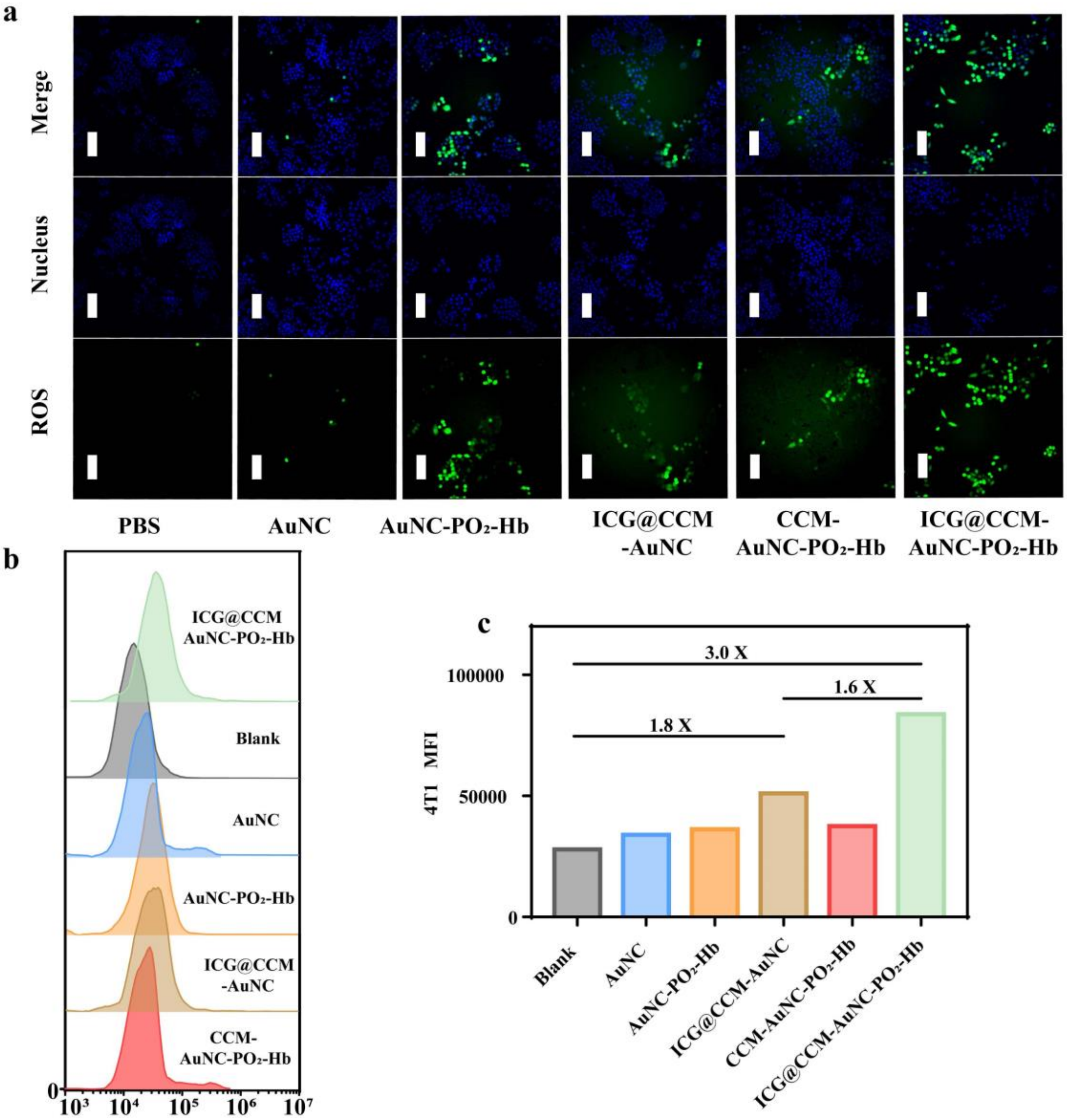

Supplementary Figure 14 Levels of intracellular ROS production. (a) ROS fluorescence images of 4T1 cells probed by DCFH-DA (Scale bar =100 μm). (b) Flow cytometry analysis of 4T1 cells after treating with ROS probe DCFH-DA and (c) its average fluorescence intensity. Experiments were performed three times (a), with similar results.

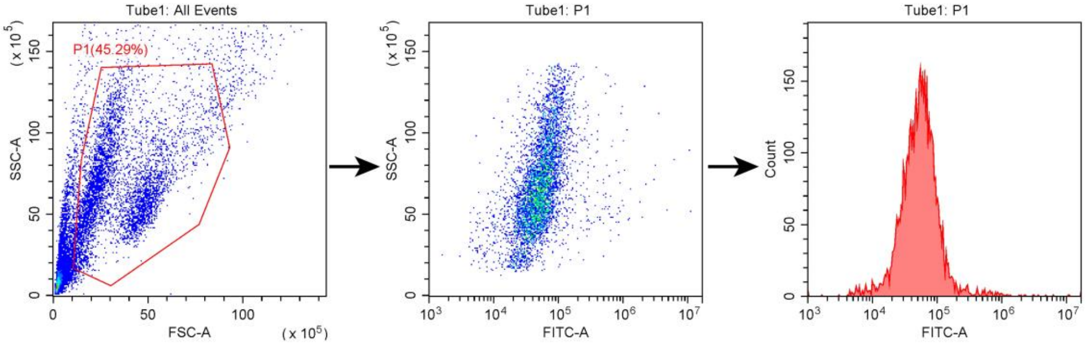

Supplementary Figure 15 Representative scatter plots and gating information derived from ROS production analysis of 4T1 cells after different treatments.

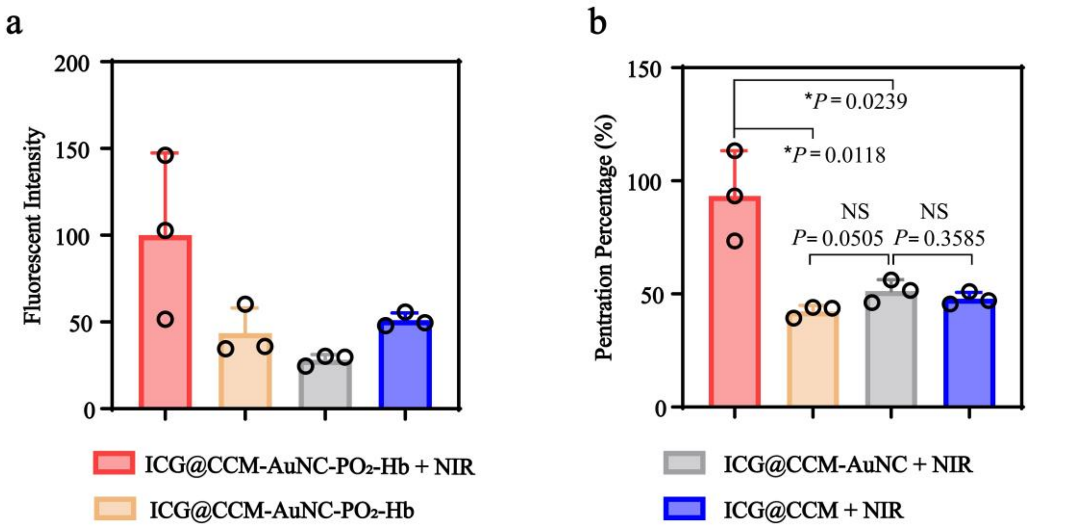

Supplementary Figure 16 Evaluation of bubble machine penetration effect. (a) Semi-quantitative intensity of tumor spheroids at 100 μm section. (b) Penetrating percentage of different formulations at 100 μm section. $n = 3$ biologically independent samples. Data are presented as means ± SD. Significant differences were evaluated by two-tailed unpaired t-test. Statistical significance: ***P*<0.01, ****P*<0.001, *****P*<0.0001, NS represented none significant differences.

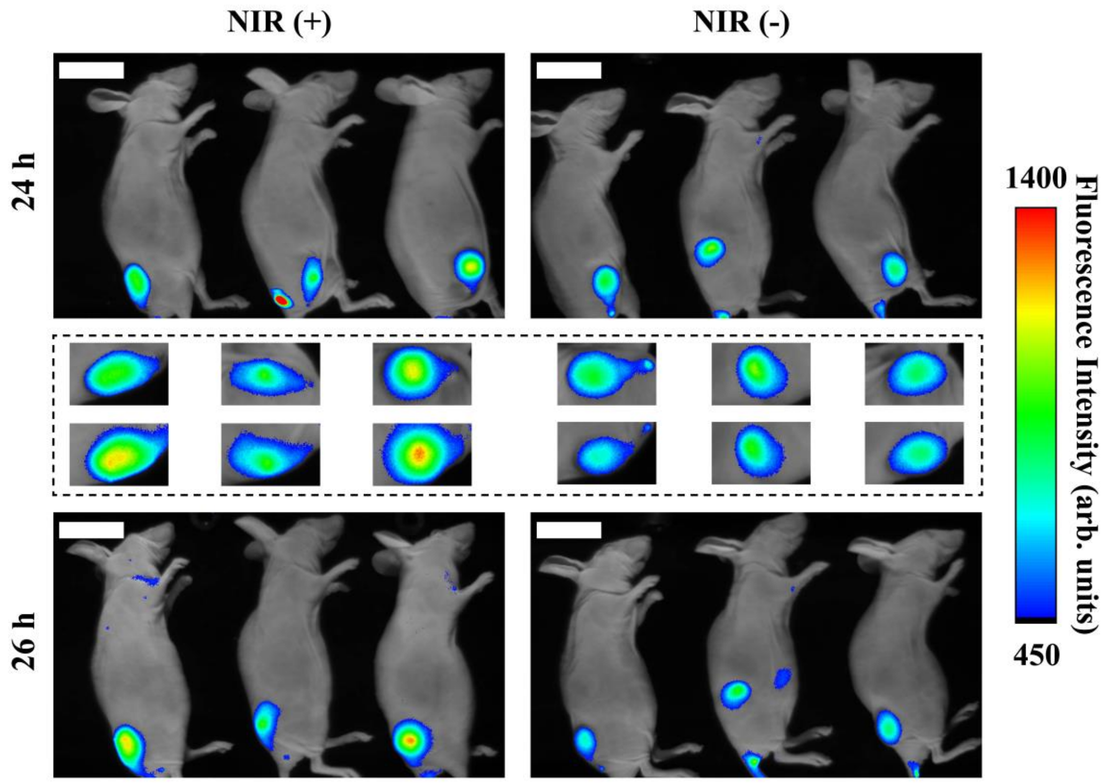

Supplementary Figure 17 Tumor penetration effects of bubble machine in mice. *In vivo* fluorescence imaging of tumor-bearing mice after ICG@CCM-AuNC-PO_2-Hb injection at time point of 24 h and 26 h. The left mice were irradiated with laser (808 nm 0.5 W cm^{-2}) for 1.5 min after first imaging at 24 h. The locally embedded maps in the middle represent the tumor sites of the corresponding mouse enlargements, respectively. Scale bars = 2 cm.

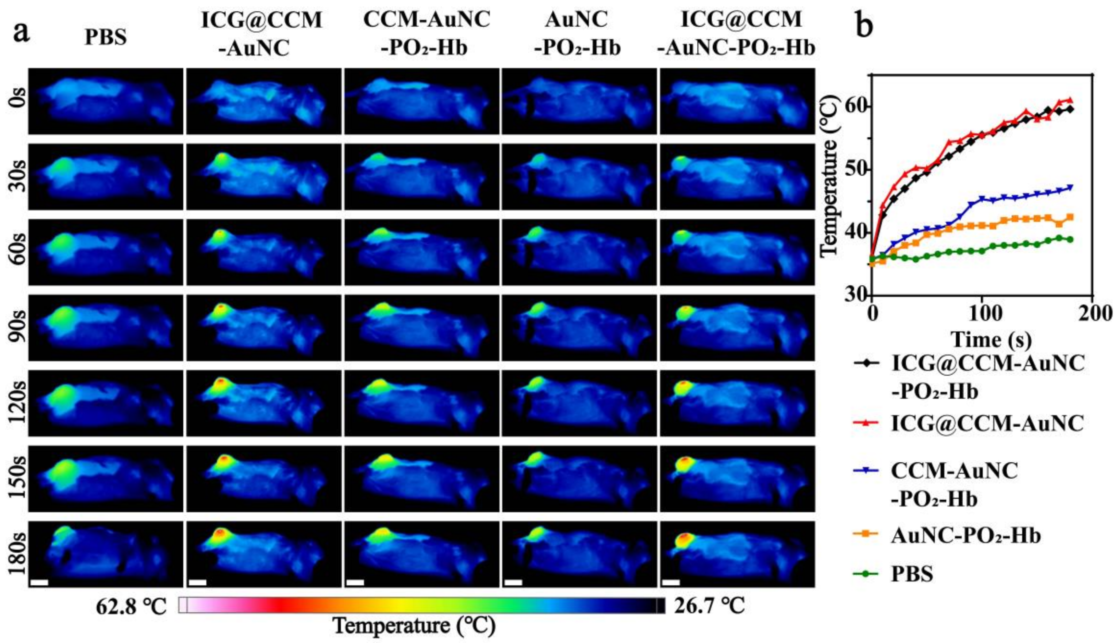

Supplementary Figure 18 Evaluation of photothermal effects *in vivo*. (a) Infrared thermography of tumor-bearing mice irradiated for 3 min (808 nm, 1.0 W cm^{-2}) after 24 h post-injection. (b) Temperature curve of the tumor from the mice during NIR irradiation. Scale bars =1 cm.

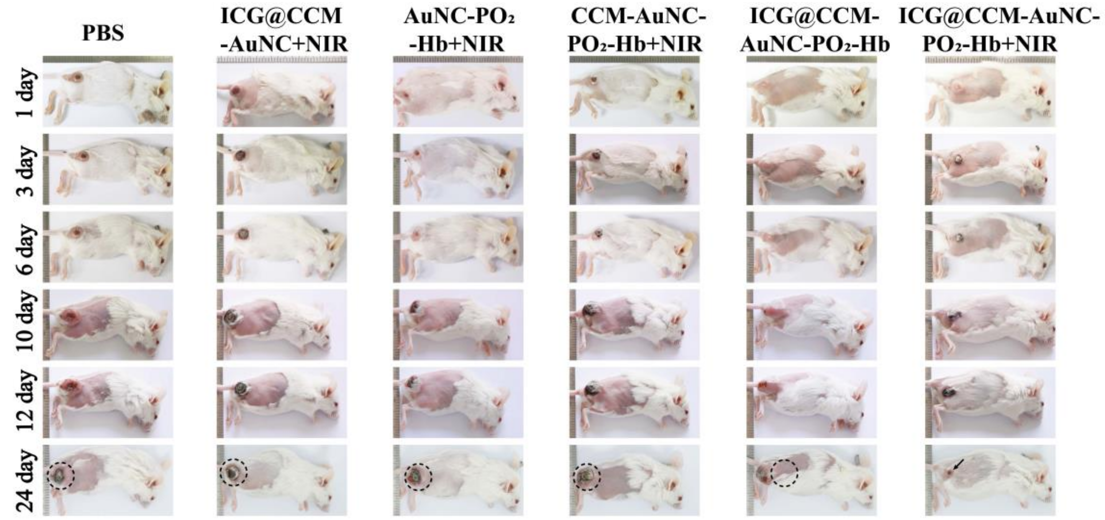

Supplementary Figure 19 Advances in the treatment of tumor bearing mice. Photographs of 4T1 tumor-bearing mice from six groups during the treatments.

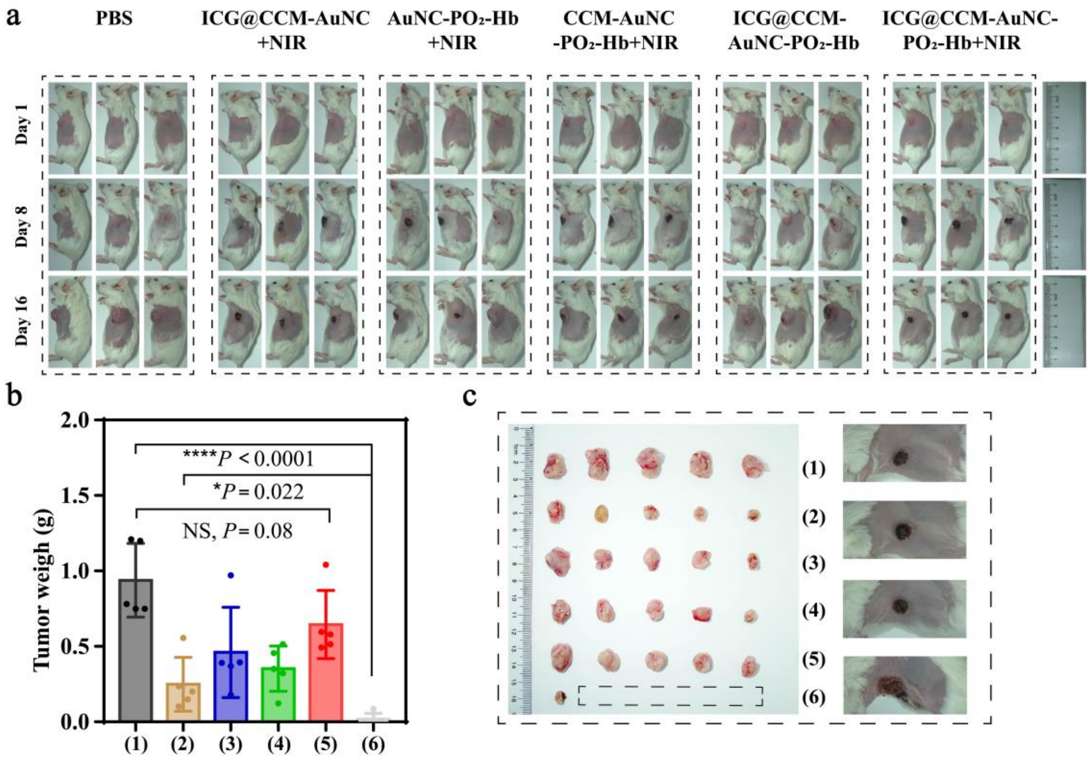

Supplementary Figure 20 The therapeutic effect of the bubble machine on orthotopic model. (a) Photographs of 4T1 tumor-bearing mice. (b) Tumor weights of the mice after administrating our bubble nanomachines (*n* = 5 biologically independent animals. Data are presented as means ± SD). Significant differences were evaluated by two-tailed unpaired t-test. (c) Collected tumors after various treatments. (1) PBS; (2) ICG@CCM-AuNC + NIR; (3) AuNC-PO_2-Hb + NIR; (4) CCM-AuNC-PO_2-Hb + NIR; (5) ICG@CCM-AuNC-PO_2-Hb; (6) ICG@CCM-AuNC-PO_2-Hb + NIR. Statistical significance: ***P*<0.01, ****P*<0.001, *****P*<0.0001, NS represented none significant differences.

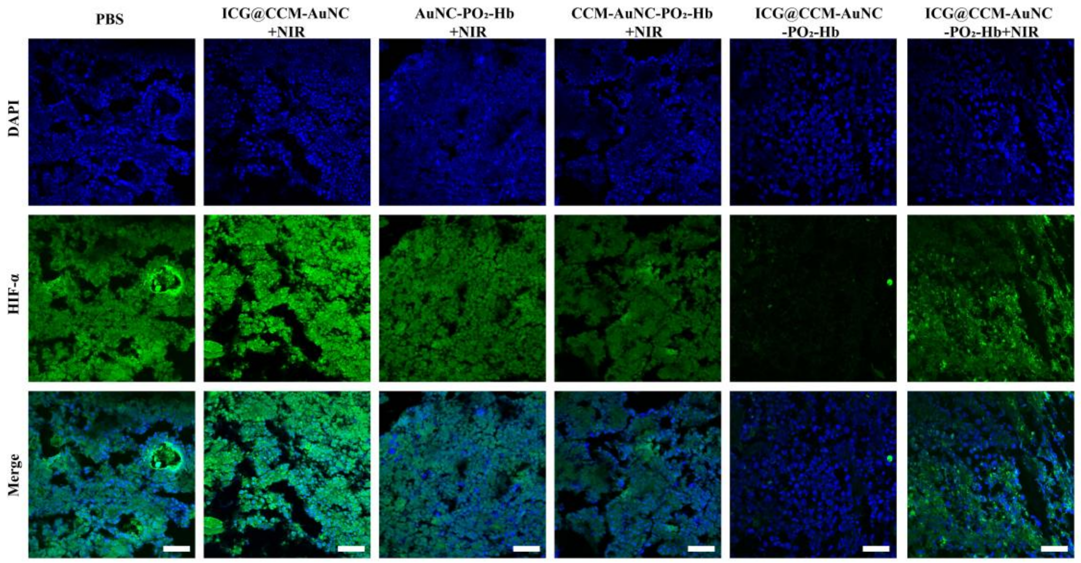

Supplementary Figure 21 Levels of ROS production *in vivo*. Immunofluorescent images of tumor slices stained with hypoxia probe. Scale bars are 50 µm. Experiments were performed three times, with similar results.

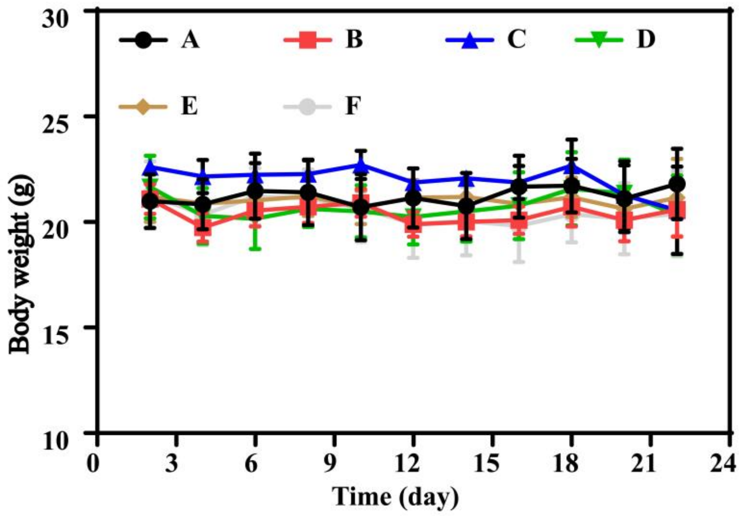

Supplementary Figure 22 Body weight changes in mice during treatment. Body weights of the mice after different treatments. (A. PBS; B. ICG@CCM-AuNC+NIR; C. AuNC-PO_2-Hb+NIR; D. CCM-AuNC-PO_2-Hb+NIR; E. ICG@CCM-AuNC-PO_2-Hb; F. ICG@CCM-AuNC-PO_2-Hb+NIR) $n = 5$ biologically independent animals. Data are presented as means ± SD.

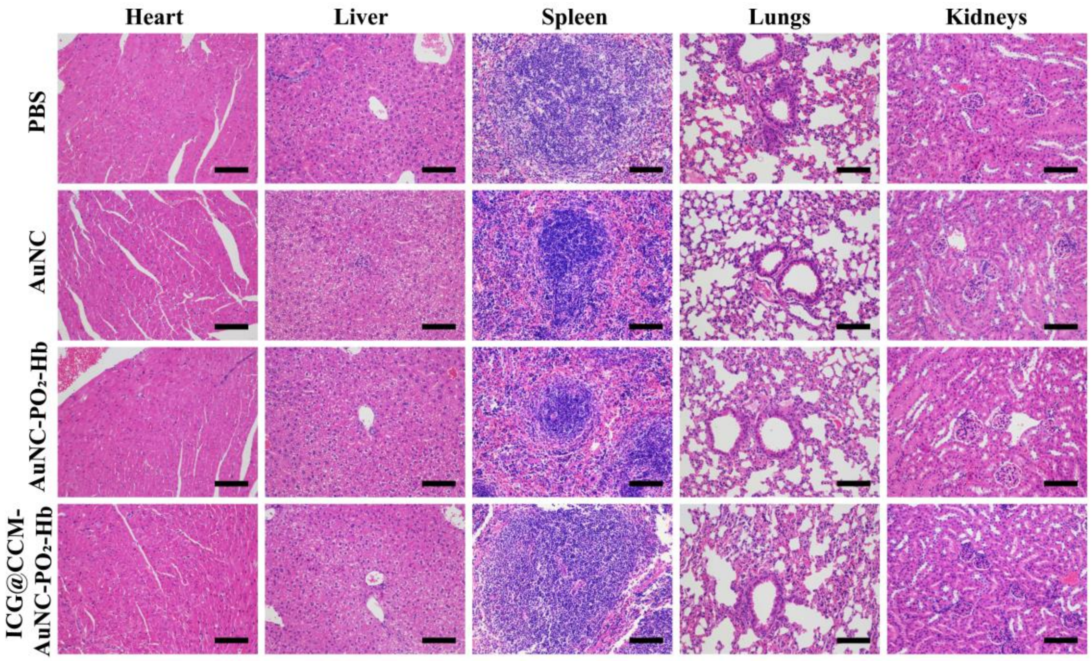

Supplementary Figure 23 *In vivo* safety evaluation. H&E staining of the heart, liver, spleen, lung and kidney tissue slices from tumor-bearing mice after treating for 24 days. Scale bar = 100 μm. Experiments were performed three times, with similar results.

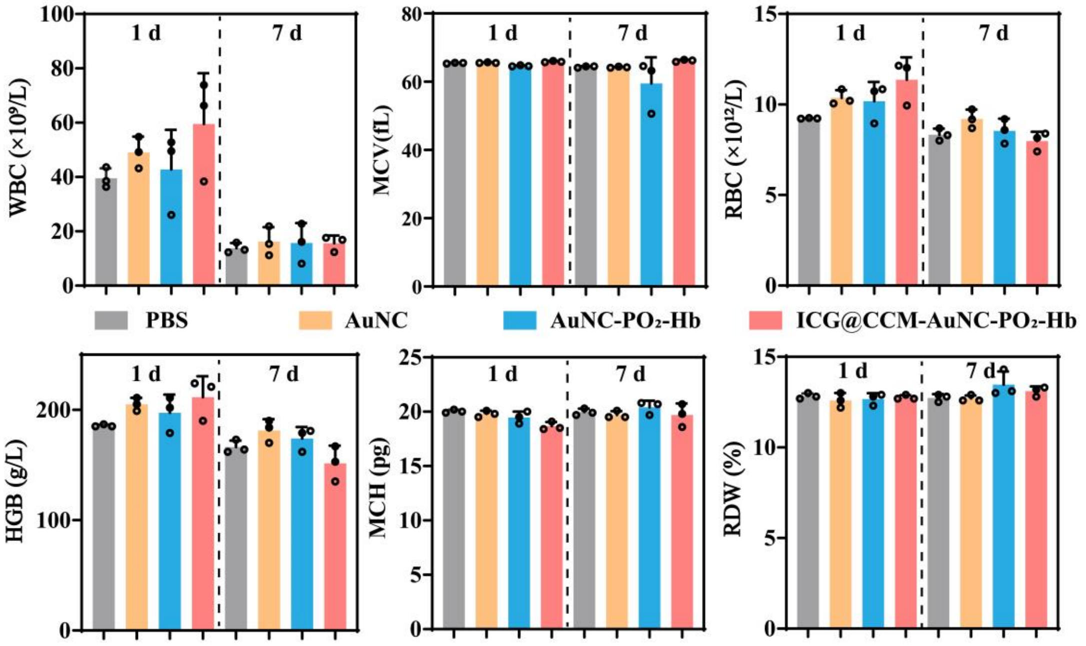

Supplementary Figure 24 Blood biochemical analysis of BALB/c mice after different treatments. $n = 3$ biologically independent samples. Data are presented as means ± SD.

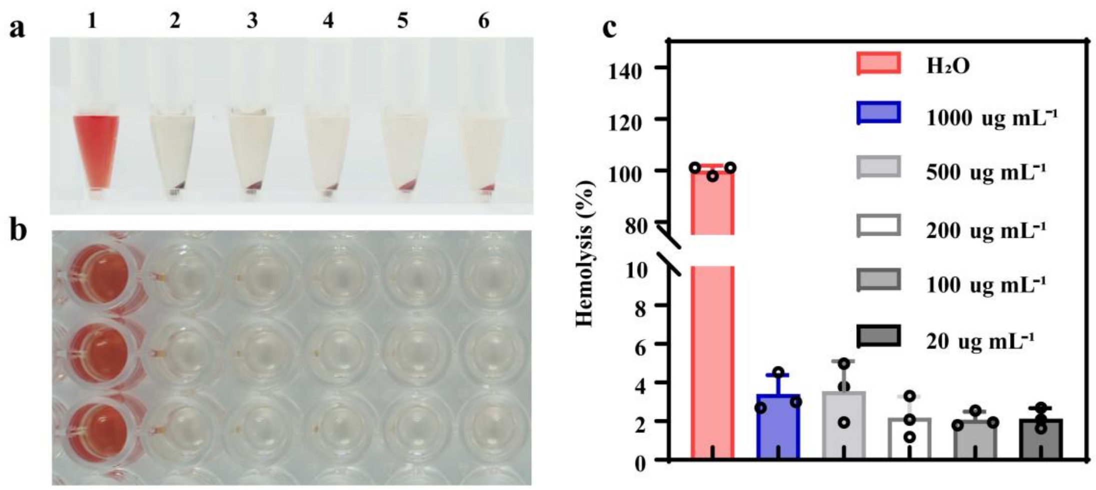

Supplementary Figure 25 Hemolytic assay of ICG@CCM-AuNC-PO_2-Hb. Hemolytic images (a) and the corresponding supernatant (b). (1: DI water, 2: 1000 μg mL^{-1}, 3: 500 μg mL^{-1}, 4: 200 μg mL^{-1}, 5: 100 μg mL^{-1}, 6: 20 μg mL^{-1}. (c) Hemolytic analysis of ICG@CCM-AuNC-PO_2-Hb. $n = 3$ biologically independent samples. Data are presented as means ± SD.
