# Supplementary material for: Hyperthermia-triggered biomimetic bubble nanomachines
Source: Nat Commun. 2023 Aug 11;14:4867. doi: 10.1038/s41467-023-40474-9 (PMC10421929; doi:10.1038/s41467-023-40474-9)
Supplement: Supplementary file 2 — Description of Additional Supplementary Files [file 41467_2023_40474_MOESM2_ESM.pdf]

Title: Supplementary Movie 1:
Description: N-SIM imaging of ICG@CCM-AuNC-PO_2-Hb during laser irradiation (808 nm, 0.5 W cm^{-2}), scale bar =1 μm.

Title: Supplementary Movie 2:
Description: N-SIM imaging of ICG@CCM-AuNC during laser irradiation (808 nm, 0.5 W cm^{-2}), scale bar =1 μm. The legends for the Supplementary Movie have been added in the manuscript.
