# Supplementary material for: Hyperthermia-triggered biomimetic bubble nanomachines
Source: Nat Commun. 2023 Aug 11;14:4867. doi: 10.1038/s41467-023-40474-9 (PMC10421929; doi:10.1038/s41467-023-40474-9)
Supplement: Supplementary file 3 — Reporting Summary [file 41467_2023_40474_MOESM3_ESM.pdf]

nature portfolio

Corresponding author(s): *Double-anonymous peer review submissions: write DAPR and your manuscript number here instead of author names.*

Last updated by author(s): *YYYY-MM-DD*

# Reporting Summary

Nature Portfolio wishes to improve the reproducibility of the work that we publish. This form provides structure for consistency and transparency in reporting. For further information on Nature Portfolio policies, see our Editorial Policies and the Editorial Policy Checklist.

## Statistics

For all statistical analyses, confirm that the following items are present in the figure legend, table legend, main text, or Methods section.

| n/a | Confirmed | |
|---|---|---|
| ☐ | ☒ | The exact sample size (*n*) for each experimental group/condition, given as a discrete number and unit of measurement |
| ☐ | ☒ | A statement on whether measurements were taken from distinct samples or whether the same sample was measured repeatedly |
| ☐ | ☒ | The statistical test(s) used AND whether they are one- or two-sided<br>*Only common tests should be described solely by name; describe more complex techniques in the Methods section.* |
| ☒ | ☐ | A description of all covariates tested |
| ☒ | ☐ | A description of any assumptions or corrections, such as tests of normality and adjustment for multiple comparisons |
| ☐ | ☒ | A full description of the statistical parameters including central tendency (e.g. means) or other basic estimates (e.g. regression coefficient) AND variation (e.g. standard deviation) or associated estimates of uncertainty (e.g. confidence intervals) |
| ☐ | ☒ | For null hypothesis testing, the test statistic (e.g. *F*, *t*, *r*) with confidence intervals, effect sizes, degrees of freedom and *P* value noted<br>*Give P values as exact values whenever suitable.* |
| ☒ | ☐ | For Bayesian analysis, information on the choice of priors and Markov chain Monte Carlo settings |
| ☒ | ☐ | For hierarchical and complex designs, identification of the appropriate level for tests and full reporting of outcomes |
| ☒ | ☐ | Estimates of effect sizes (e.g. Cohen's *d*, Pearson's *r*), indicating how they were calculated |

*Our web collection on statistics for biologists contains articles on many of the points above.*

## Software and code

Policy information about availability of computer code

| | |
|---|---|
| Data collection | TEM (Tecnai G2 F30, FEI), Uv-vis-NIR (UV-2600, shimadzu), Infrared Thermal Camera (Ti480 pro, Fluke), Confocal Microscopy (LSM 880, CARL Zeiss), Flucorescence Microscope (Ti2-A, Nikon), Flow Cytometry (cytoFLEX, Beckmen), SEM (ProX , Phenom), Nanoparticle Tracking Analysis (PMX, Zetaview), Fluorescence spectra (RF-6000, Shimadzu), Near Inrared irradiation (Stone), In Vivo Imaging (In-Vivo FX PRO, Bruker), Circular Dichroism (Chirascan, Applied Photophysics), Inductively Coupled Plasma-Mass Spectrometry (ICP-MS) (Thermo Fisher Scientific). |
| Data analysis | All statistical analyses were analyzed by Microsoft office Excel 2019, Graphpad Prism 7 and image J (1.52a). Flow cytometry data were analyzed on CytExpert 1.2 software package. Living image software (Bruker MI) was used to analyse fluorescent images. The secondary structure of hemoglobin was computed by DichroWeb online analysis. |

For manuscripts utilizing custom algorithms or software that are central to the research but not yet described in published literature, software must be made available to editors and reviewers. We strongly encourage code deposition in a community repository (e.g. GitHub). See the Nature Portfolio guidelines for submitting code & software for further information.

## Data

Policy information about availability of data

All manuscripts must include a data availability statement. This statement should provide the following information, where applicable:

- Accession codes, unique identifiers, or web links for publicly available datasets
- A description of any restrictions on data availability
- For clinical datasets or third party data, please ensure that the statement adheres to our policy

The source data generated in this study are provided in the Supplementary Information/Source Data file. The full image dataset is available from the corresponding author upon request. Source data are provided with this paper.

## Research involving human participants, their data, or biological material

Policy information about studies with human participants or human data. See also policy information about sex, gender (identity/presentation), and sexual orientation and race, ethnicity and racism.

| | |
|---|---|
| Reporting on sex and gender | N/A |
| Reporting on race, ethnicity, or other socially relevant groupings | N/A |
| Population characteristics | N/A |
| Recruitment | N/A |
| Ethics oversight | N/A |

Note that full information on the approval of the study protocol must also be provided in the manuscript.

# Field-specific reporting

Please select the one below that is the best fit for your research. If you are not sure, read the appropriate sections before making your selection.

☒ Life sciences ☐ Behavioural & social sciences ☐ Ecological, evolutionary & environmental sciences

For a reference copy of the document with all sections, see nature.com/documents/nr-reporting-summary-flat.pdf

# Life sciences study design

All studies must disclose on these points even when the disclosure is negative.

| | |
|---|---|
| Sample size | No statistical methods was used to predetermine the samples size. The sample sizes were determined as minimal to lower the cost and be sufficient to obtain statistically significant difference between experimental groups(n=3-5). For property measurement experiments, samples were prepared and tested at least twice. For in vivo studies, each group contains at least 3 for evaluating the statistical significance. These sample sizes also represent the standard practice for publication in this field and were described in figure legends. Each sample represents independent biological replicates. |
| Data exclusions | No data were excluded. |
| Replication | All samples were replicated independently for 2-3 times with similar results. All attempts at replication were successful. |
| Randomization | The samples were randomly grouped. |
| Blinding | The investigators were blinded to the group allocation during these studies. Analyses in the animal experiments were based on measurements acquired and mostly performed by the same investigator. |

# Reporting for specific materials, systems and methods

We require information from authors about some types of materials, experimental systems and methods used in many studies. Here, indicate whether each material, system or method listed is relevant to your study. If you are not sure if a list item applies to your research, read the appropriate section before selecting a response.

## Materials & experimental systems

| n/a | Involved in the study | |
|---|---|---|
| ☐ | ☒ | Antibodies |
| ☐ | ☒ | Eukaryotic cell lines |
| ☒ | ☐ | Palaeontology and archaeology |
| ☐ | ☒ | Animals and other organisms |
| ☒ | ☐ | Clinical data |
| ☒ | ☐ | Dual use research of concern |
| ☒ | ☐ | Plants |

## Methods

| n/a | Involved in the study | |
|---|---|---|
| ☒ | ☐ | ChIP-seq |
| ☐ | ☒ | Flow cytometry |
| ☒ | ☐ | MRI-based neuroimaging |

# Antibodies

Antibodies used

Donkey Anti-Rabbit IgG H&L (Alexa Fluor 647), (ab150075, Abcam, 1:500)
CD44 Polyclonal Antibody, (15675-1-AP, Proteintech,1:200)
Anti-HIF-1 alpha antibody, (ab179483, Abcam, 1:200)
Goat pAb to Rb IgG (Alexa Fluor 488), (ab150077, Abcam, 1:500)

Validation

All antibodies were verified by the supplier and each lot has been quality tested. All the antibodies used are from commercial sources and have been validated by the vendors. Validation data are available on the manufacturer's website.
1. Donkey Anti-Rabbit IgG H&L (Alexa Fluor 647)
https://www.abcam.cn/products/secondary-antibodies/donkey-rabbit-igg-hl-alexa-fluor-647-ab150075.html
2. CD44 Polyclonal Antibody
https://www.ptgcn.com/products/CD44-Antibody-15675-1-AP.htm
3. Recombinant Anti-HIF-1 alpha antibody
https://www.abcam.cn/products/primary-antibodies/hif-1-alpha-antibody-epr16897-ab179483.html
4. Goat pAb to Rb IgG (Alexa Fluor 488)
https://www.abcam.cn/products/secondary-antibodies/goat-rabbit-igg-hl-alexa-fluor-488-ab150077.html

# Eukaryotic cell lines

Policy information about cell lines and Sex and Gender in Research

Cell line source(s)

NIH3T3 (Mouse embryonic fibroblast) cells were kindly provided by Dr. Weichang Huang from The First Clinical Medical College of Southern Medical University.
B16-F10 (Mouse melanoma) cells were obtained from the ATCC (CRL-6475).
4T1 (Mouse mammary tumor) cells (Lot: XRBETC2TZM) were purchased from Procell Life Science&Technology Co.,Ltd.

Authentication

The cell line we used was morphologically confirmed according to the information provided by SIBS.

Mycoplasma contamination

All cells were tested to be free of mycoplasma contamination.

Commonly misidentified lines
(See ICLAC register)

No cell lines used are listed in the database of commonly misidentified cell lines.

# Animals and other research organisms

Policy information about studies involving animals; ARRIVE guidelines recommended for reporting animal research, and Sex and Gender in Research

Laboratory animals

Female BALB/c mice (4-6 weeks old, 18-22g) and BALB/c nude mice (5-6 weeks old, 17-20g) were obtained from the Laboratory Animal Center of Southern Medical University. The mice had access to food and water ad libitum and were hosted in ambient temperature (22–24 °C), humidity at 30–70%, under 12 h dark/light cycles.

Wild animals

The study did not involve wild animals.

Reporting on sex

In terms of animal experiment studies, female mice were chosen.

Field-collected samples

This study did not involve samples collected from field.

Ethics oversight

All the animal procedures were carried out under the guideline approved by the Institutional Animal Care and Use Committee (IACUC) of Southern Medical University (permit number: SMUL2022180).

Note that full information on the approval of the study protocol must also be provided in the manuscript.

## Flow Cytometry

### Plots

Confirm that:

- [x] The axis labels state the marker and fluorochrome used (e.g. CD4-FITC).
- [x] The axis scales are clearly visible. Include numbers along axes only for bottom left plot of group (a 'group' is an analysis of identical markers).
- [x] All plots are contour plots with outliers or pseudocolor plots.
- [x] A numerical value for number of cells or percentage (with statistics) is provided.

### Methodology

| | |
|---|---|
| Sample preparation | For the homologous targeting analysis, B16-F10 cells/ NIH3T3 cells and 4T1 cells were collected after treatments and washed with PBS, then analyze the fluorescence signal.<br>For intracellular ROS detection, the 4T1 cells were collected after treatments and incubated with DCFH-DA (10 μM) for 30 min. The cell were then irradiated with NIR laser (808 nm, 1.0 W cm-2) for 2 min and washed with PBS, finally analyze the fluorescence level. |
| Instrument | Flow Cytometry (cytoFLEX, Beckmen) |
| Software | Data acquired and analyzed using CytExpert 1.2 software. |
| Cell population abundance | No sorting was performed. |
| Gating strategy | For all experiments, the gate was set to "viable" cells in FSC/SSC plot, and from those populations of live cells were gated. |

- [x] Tick this box to confirm that a figure exemplifying the gating strategy is provided in the Supplementary Information.
